# Fibrinogen Is Associated with Prognosis of Critically Ill Patients with Sepsis: A Study Based on Cox Regression and Propensity Score Matching

**DOI:** 10.1155/2023/7312822

**Published:** 2023-03-20

**Authors:** Chi Yao, Guangyuan Zhang, Nieke Zhang, Renjie Li, Si Sun, Lei Zhang, Yi Xia, Shuqiu Chen, Jin Sun, Ming Chen

**Affiliations:** ^1^Department of Urology, Zhongda Hospital, Southeast University, Nanjing 210009, China; ^2^School of Medicine, Southeast University, Nanjing 210009, China; ^3^Department of Urology, Xuyi People's Hospital, Huaian 211799, China

## Abstract

**Introduction:**

Sepsis is a common syndrome in critically ill patients. Fibrinogen was reported to be associated with the prognosis of sepsis patients.

**Materials and Methods:**

Data was acquired from Multiparameter Intelligent Monitoring in Intensive Care Database IV (MIMIC-IV) version 1.0. Cox proportional hazards regression was utilized to estimate the relationship between fibrinogen and inhospital mortality. The cumulative incidence of mortality by fibrinogen level was estimated through the Kaplan-Meier curve. Restricted cubic spline (RCS) was used to assess nonlinear relationship. Subgroup analysis was also conducted to evaluate the robustness of the association between fibrinogen and inhospital mortality. Propensity score matching (PSM) was applied to adjust for confounding factors.

**Results:**

A total of 3365 patients, including 2031 survivors and 1334 nonsurvivors, were enrolled in our study. The survivors had a significantly elevated levels of fibrinogen compared with the deceased. The elevated level of fibrinogen was significantly associated with a decrease in mortality in multivariate Cox regression before and after PSM (HR 0.66, *P* < 0.001 and HR 0.73, *P* < 0.001, respectively). RCS showed a nearly linear relationship. Subgroup analysis demonstrated the robustness of the association in most subpopulations. However, the association between decreased levels of fibrinogen and increased inhospital mortality was denied after PSM.

**Conclusion:**

The elevated level of fibrinogen hints at better overall survival in critically ill patients with sepsis. Decreased levels of fibrinogen may be of little value in identifying patients with a high risk of death.

## 1. Introduction

Sepsis is a common syndrome that occurs when an infection leads to a systemic inflammatory response and organ dysfunction [1]. It is reported that sepsis impacted millions of people worldwide in a year and killed between one in three and one in six patients suffering from it [2–4]. Coagulopathy usually accompanies sepsis, which is mainly attributed to intravascular activation of coagulation and microvascular endothelial injury [5]. Some hemostasis biomarkers are claimed to be risk factors for attacks and death from sepsis [6, 7].

Fibrinogen is a serum glycoprotein that serves as an important coagulation factor and also gets involved in the inflammation response, like regulation of macrophage adhesion and activation of cytokine/chemokine production [8–10]. It has been demonstrated that a decreased level of fibrinogen is significantly associated with an increase in mortality and an elevated level of fibrinogen makes little difference to the prognosis [11]. Additionally, children with fibrinogen levels lower than 2 g/L were considered to be at a greater risk of hospital death [12].

Here, we conducted a retrospective study to further investigate the association between fibrinogen and in-hospital mortality of sepsis patients by the Cox proportional hazards regression model and propensity score matching (PSM). Subgroup analysis and restricted cubic spline (RCS) were also carried out to reveal the robustness and evaluate the nonlinear relationship.

## 2. Materials and Methods

### 2.1. Data Source

Data was acquired from the Multiparameter Intelligent Monitoring in Intensive Care Database IV (MIMIC-IV) version 1.0 which enrolled more than 60000 ICU stays between 2008 and 2019. The database was operated by the Beth Israel Deaconess Medical Center [13]. We accomplish the course, Protecting Human Research Participants (certification number: 46538344), which is a National Institutes of Health web-based course. Our permission was approved by the Institutional Review Boards of the Massachusetts Institute of Technology (Cambridge, MA, USA) and the Beth Israel Deaconess Medical Center.

### 2.2. Population Selection

Patients were enrolled if they met the following criteria: (1) diagnosed with sepsis according to Sepsis-3 standards [1]; (2) age ≥ 18 years; (3) stay in ICU for more than 24 hours; (4) complete medical records and laboratory data; and (5) first stay was reserved if repetitively admitted into ICU.

### 2.3. Data Extraction

We utilized Structured Query Language (SQL) to acquire information recorded on the first day after ICU admission. The gender and age of patients, treatment like the use of ventilation and renal replacement therapy (RRT), the Simplified Acute Physiology Score (SAPS) II and the Sequential Organ Failure Assessment (SOFA) score, laboratory tests including fibrinogen, white blood cells (WBC), serum creatine (Scr), hemoglobin, albumin, platelets, potassium, sodium, lactate, total bilirubin, glucose, prothrombin time (PT) and partial thromboplastin time (PTT), comorbidities including acute kidney injury (AKI), acute myocardial infarction (AMI), heart failure (HF), liver cirrhosis, hypertension, chronic obstructive pulmonary disease (COPD), chronic kidney disease (CKD), atrial fibrillation (AF), cerebral infarction, cerebral hemorrhage, thrombosis, and acute respiratory failure. All these data stated above were extracted and can be seen in Table 1.

### 2.4. Statistical Analysis

Continuous variables of normal distribution were shown as mean ± standard deviation (SD). Categorical variables were presented as frequency (percent). A one-way ANOVA or student *t*-test was applied to normally distributed continuous variables to determine the difference between groups. The Kruskal–Wallis test was utilized in nonnormally distributed continuous variables, and the Chi-square test was used in categorical data.

Cox proportional hazards regression model and propensity score matching were applied in our study. In the Cox model, patients with fibrinogen were stratified into 3 groups: [1] low level<150 mg/dL; [2] normal level 150-400 mg/dL; and [3] high level>400 mg/dL. The quantitative value of laboratory tests was transformed to be categorical data by the lower and upper limits of the normal range. Specifically, creatine, hemoglobin, albumin, lactate, and bilirubin were categorized to be binary variables. WBC, platelets, potassium, sodium, blood glucose, PT, and PTT were classified into 3 levels.

The hazard ratio (HR) with 95% confidence intervals (CIs) was calculated to estimate the relationship between fibrinogen and inhospital mortality. Patients with normal fibrinogen were used as the reference group in the Cox regression. We established 3 multivariate COX models. Model 1 adjusts for age and gender. Model 2 adjusts for model 1 plus comorbidities including AKI, AMI, HF, liver cirrhosis, hypertension, COPD, CKD, AF, cerebral infarction, cerebral hemorrhage, thrombosis, and acute respiratory failure. Model 3 adjusts for model 2 plus WBC, Scr, hemoglobin, albumin, platelets, blood potassium, sodium, lactate, total bilirubin, glucose, prothrombin time (PT and PTT), use of ventilation, and RRT. Subgroup analysis was conducted to further assess the association between fibrinogen and inhospital mortality. The effect modification of other factors on fibrinogen in multivariate Cox regression was also evaluated by calculating *P* for interaction. The cumulative incidence of mortality by fibrinogen level was estimated through the Kaplan-Meier curve.

To further confirm the robustness of the correlation, we utilized the nearest PSM with a ratio of 1 : 1 and a caliper of 0.005. Patients with fibrinogen<150 mg/dL and patients with fibrinogen>400 mg/dL match patients with normal fibrinogen, respectively. After matching, the difference in mortality was assessed by Chi-square and COX regression model again. Moreover, the nonlinear relationship between fibrinogen and inhospital mortality was assessed using restricted cubic spline curves.

All statistical analyses were performed using R V.3.6.3 (R Foundation for Statistical Computing, Vienna, Austria) and Stata 16.0 (StataCorp LLC, Texas, USA).

## 3. Results

### 3.1. Population and Baseline Characteristics

A total of 3365 eligible sepsis individuals were identified. The characteristics of enrolled patients are shown in Table 1. In our cohorts, 2031 patients survived and 1334 patients ended in death. The survivors had a significantly elevated levels of fibrinogen and a reduced levels of creatine, lactate, and bilirubin compared with the deceased. The rate of AMI, liver cirrhosis, atrial fibrillation, acute respiratory failure, and AKI was significantly higher in the deceased than in survivors.

**Figure 1:**
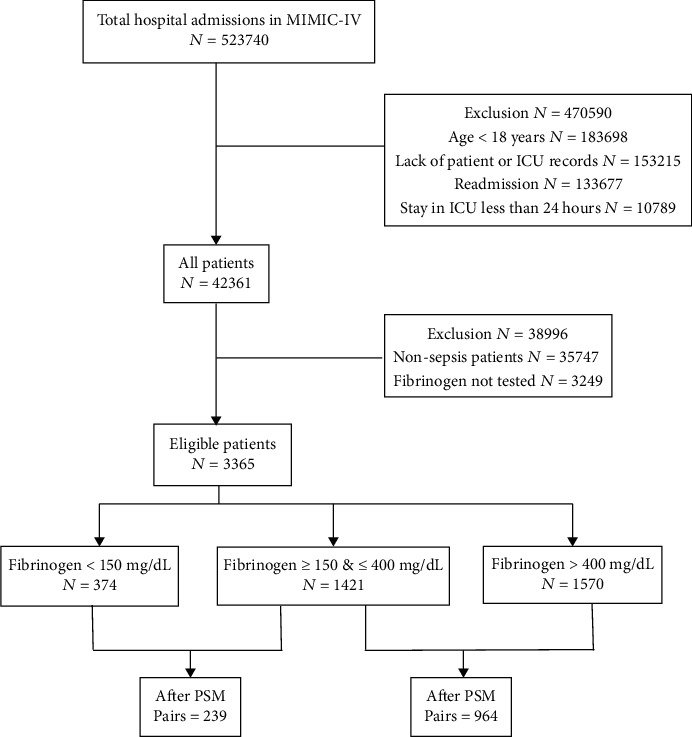
Flowchart of patient selection.

In propensity score matching of the decreased fibrinogen cohort and the normal fibrinogen cohort, 239 pairs of patients succeeded in matching. As to the matching of the elevated fibrinogen cohort and the normal fibrinogen cohort, 964 pairs of patients were matched. The characteristics of matched patients are balanced, as shown in Tables 2 and 3. The flowchart of patient selection can be seen in Figure 1.

### 3.2. Fibrinogen and Inhospital Mortality

The aggregation of univariate and multivariate Cox regression can be seen in Table 4. In univariate Cox regression, age, the presence of AMI, liver cirrhosis, COPD, CKD, atrial fibrillation, acute respiratory failure and AKI, the decreased level of fibrinogen, hemoglobin, albumin, platelet and sodium, increased level of creatine, bilirubin, potassium, sodium, PT, and APTT were considered risk factors for inhospital mortality. The increased level of fibrinogen was the only protective factor identified in univariate Cox regression. In the multivariate Cox regression model, age, the presence of acute respiratory failure and AKI, the decreased level of fibrinogen, WBC, creatine, hemoglobin, sodium and glucose, the increased level of creatine, sodium, lactate, and APTT remain risk factors. As to the protective factor, the elevated level of fibrinogen stands alone.

Additionally, the hazard ratios of fibrinogen on inhospital mortality in different Cox models are summarized in Table 5. An elevated level of fibrinogen is always a protective factor and decreased level of fibrinogen remain a risk factor in different models.

**Figure 2:**
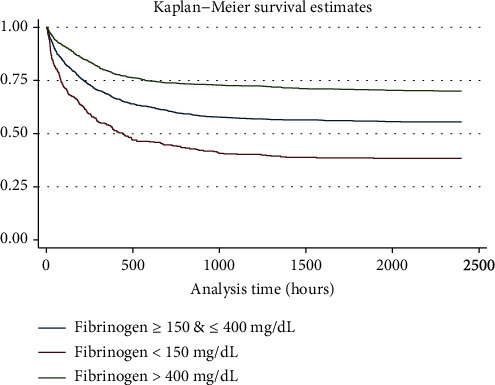
Kaplan-Meier survival curve.

Moreover, the Kaplan-Meier survival curve showed a significantly lower survival rate in decreased fibrinogen group and a higher survival rate in the elevated fibrinogen group compared with the normal fibrinogen group (Figure 2).

### 3.3. Subgroup Analysis and *P* for Interaction

Most subpopulations showed similar hazard ratios concerning the association between fibrinogen and inhospital mortality in adjusted model 3. Atrial fibrillation, acute respiratory failure, WBC level, and bilirubin level interacted significantly with fibrinogen level (Table 6).

### 3.4. Propensity Score Matching

**Figure 3:**
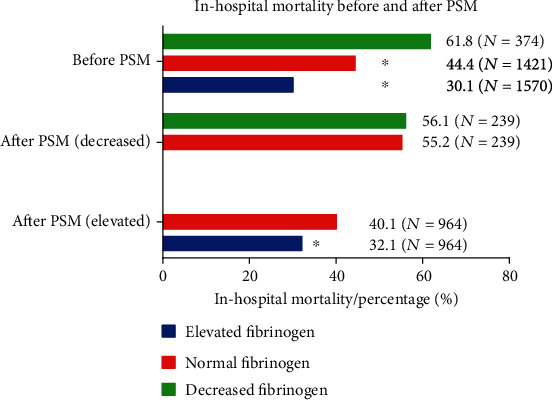
A brief summary of inhospital mortality before and after PSM. ^∗^*P* < 0.001 in the Chi-square test.

Statistical difference of inhospital mortality between an elevated fibrinogen (>400 mg/dL) group and the normal fibrinogen group (150-400 mg/dL) remains (normal level vs. elevated fibrinogen: 40.1% vs. 32.1%, *P* < 0.001). Conversely, the difference between decreased fibrinogen (<150 mg/dL) and normal fibrinogen group (44.4% vs 61.8%) vanished after PSM (55.2% vs. 56.1%). A brief summary of inhospital mortality can be seen in Figure 3. Specific information can be seen in Tables 2 and 3.

After PSM, another multivariate Cox regression was conducted based on data from the elevated fibrinogen cohort and normal fibrinogen cohort. The HR with 95% confidence intervals was 0.73 (0.63, 0.85) (*P* < 0.001), changing a little from 0.66 (0.59, 0.75) (*P* < 0.001) before PSM.

**Figure 4:**
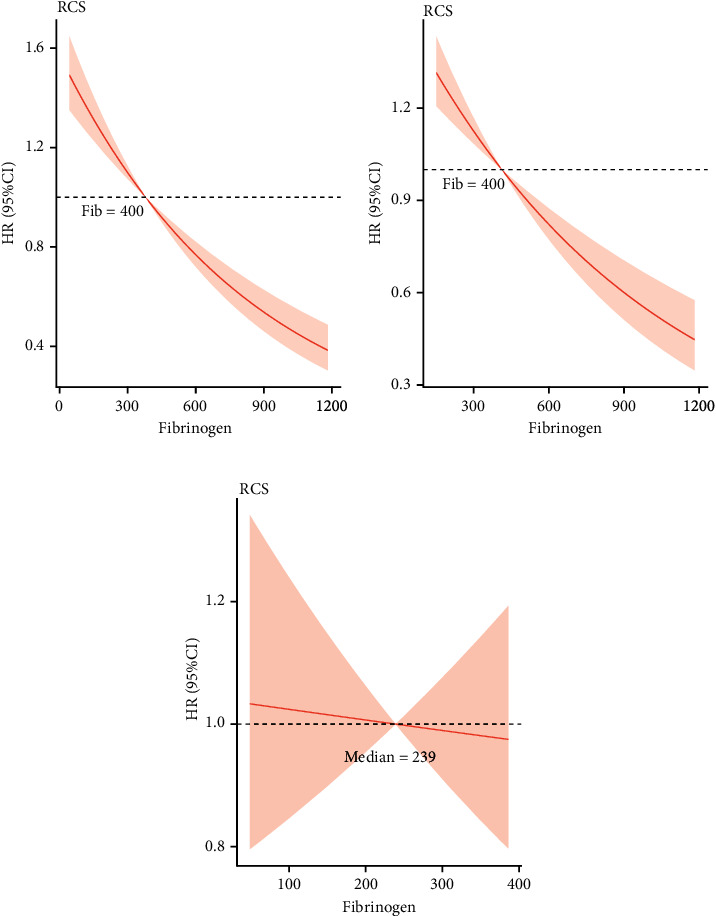
Restricted cubic spline of (a) Cox regression based on all included patients before propensity score matching. (b) Cox regression based on patients with elevated fibrinogen and normal fibrinogen after PSM. (c) Cox regression based on patients with decreased and normal fibrinogen after PSM.

To further investigate the relationship between fibrinogen and inhospital mortality, restricted cubic spline (RCS) curves before and after PSM were plotted. A nearly linear relationship is demonstrated in (Figure 4).

## 4. Discussion

The retrospective study further revealed that fibrinogen levels are closely associated with the clinical outcome of sepsis patients. The Cox regression suggested that a decreased level of fibrinogen is a risk factor for inhospital mortality and elevated level of fibrinogen is significantly associated with a reduction in inhospital death. However, the difference in mortality between patients with decreased fibrinogen and patients with normal fibrinogen is eliminated after adjustment using propensity score matching, which is inconsistent with the result of Cox model and previous studies [11, 12]. Moreover, we first reported that elevated level of fibrinogen reminds better overall survival in critically ill patients with sepsis.

Sepsis is a leading cause of mortality and critical illness globally [2, 14]. Inflammation and coagulation played complementary roles to defend the host from infection, and both of them were involved in tissue damage in the early stage of sepsis [15, 16]. As a key component in the coagulation cascade, fibrinogen converts to fibrin when inflammation happens [17]. Furthermore, fibrinogen will facilitate leukocyte transmigration out of vessels and evoke leukocyte effector functions [17, 18]. Simultaneously, plasma fibrinogen will increase immediately as an acute response to inflammation, which was reported to preserve until the end-stage of sepsis [19, 20]. From the perspective of clinicians, abnormal plasma fibrinogen indicates the consumption of hemostatic factors, excessive hypercoagulation, and hyperfibrinolysis in sepsis [21].

Our study recognized elevated levels of fibrinogen as a protective factor for sepsis patients in both the multivariate Cox regression models and the restricted cubic spline, which was further demonstrated using PSM. Nearly all patients with sepsis have coagulation abnormalities, which may progress to disseminated intravascular coagulation (DIC) [22, 23], a severe coagulopathy usually characterized by depleting platelets and fibrinogen and a high probability of death [5]. Thus, it is reasonable to assume that an elevated level of fibrinogen hints at the favorable compensation of the human body and avoidance of the slide into DIC, and even predicts better survival than patients with normal fibrinogen. Honestly, we did not explore the causality of fibrinogen and inhospital mortality elaborately with statistical methods like marginal structural modeling, which plays a key role in estimating the effects of potential confounders, especially longitudinal data [24]. However, as stated above, we are inclined to consider elevated or decreased levels of fibrinogen as a response to sepsis, instead of an exposure or intervention. Such a detectable response or alteration of biomarker just reminds the prognosis of patients with sepsis. Additionally, we cannot rule out the possibility that elevated levels of fibrinogen may play a role in modulating inflammation induced in sepsis, which even reminds more radical and aggressive administration of fibrinogen in sepsis patients in clinical practice. We tried to verify the positive effects of intravenous infusion of fibrinogen in sepsis patients, but in vain due to the lack of records in the database. Further animal experiments are conducted to authenticate or disprove the inference in our laboratory.

Propensity score matching (PSM) was reported to be capable of reducing or even eliminating the effects of selection bias in prospective and retrospective study [25, 26]. Despite the inconsistency with the initial outcome in our Cox regression model, our analysis of paired samples after PSM casts doubt on the authenticity of the statement that decreased level of fibrinogen was associated with an increase in mortality [11]. A summary comparing the results of regression before and after PSM can be seen in Supplementary Table 1. Logically, PSM makes our opposite conclusion more persuasive with the reduction of bias. Specifically, PSM could reduce the differences in illness severity and basic physical condition between groups by matching the SOFA score, SAPS II, and laboratory tests like albumin and hemoglobin. Admittedly, based on merely 239 pairs of patients, it is exaggerated to overturn the conclusion in the previous multicenter cohort study [11], which is also confirmed in critically ill children [12, 27]. Anyhow, further unbiased studies are necessary to dissolve the controversy.

AKI was also an independent prognostic factor for inhospital mortality in critically ill sepsis patients in our multivariate Cox regression model. As individual syndromes, sepsis and acute kidney injury (AKI) render the host susceptible to each other [28, 29]. Usually, sepsis-associated acute kidney injury (S-AKI) is defined as AKI in the presence of sepsis excluding other etiological factors of AKI or satisfying criteria of both sepsis-3 and kidney disease: improving global outcomes (KDIGO) [30, 31]. Moreover, sepsis-associated acute kidney injury (S-AKI) is reported to be common in critically ill patients, which leads to a dramatically increased risk of chronic comorbidities and mortality [32, 33]. In our study, we do not distinguish S-AKI from overall AKI. Despite the fact that we ignore the subdivision of AKI, the presence of AKI still reveals a worse prognosis, which corresponds with previous studies.

There are several limitations in the study. First, we extracted the initial value of fibrinogen upon admission to ICU and ignore the exogenous administration of them before and during the ICU stay, which may exert positive or negative effects. Second, the data were acquired from a single central database. Though inclusion standards were set, selection bias was somewhat unavoidable. Finally, merely a relatively small sample, 239 pairs of patients, was kept for analysis of patients with decreased fibrinogen after PSM, which makes our statement less persuasive.

## 5. Conclusions

The elevated level of fibrinogen hints at better overall survival in critically ill patients with sepsis. Decreased levels of fibrinogen may be of little value in identifying patients at high risk of death.

## Figures and Tables

**Table 1:** Baseline characteristics of enrolled patients.

Characteristics	Total (*n* = 3365)	Survivors (*n* = 2031)	Nonsurvivors (*n* = 1334)	*P*
Age, years	63.31 (16.41)	61.46 (16.93)	66.12 (15.17)	<0.001
Gender, *n* (%)	1880 (55.90)	1149 (56.6)	731 (54.8)	0.328
Comorbidities, *n* (%)				
AKI	2355 (70.0)	1283 (63.2)	1072 (80.4)	<0.001
AMI	197 (5.9)	92 (4.5)	105 (7.9)	<0.001
Heart failure	990 (29.4)	562 (27.7)	428 (32.1)	0.007
Liver cirrhosis	562 (16.7)	256 (12.6)	306 (22.9)	<0.001
Hypertension	681 (20.2)	415 (20.4)	266 (19.9)	0.761
COPD	239 (7.1)	125 (6.2)	114 (8.5)	0.01
CKD	752 (22.3)	421 (20.7)	331 (24.8)	0.006
Atrial fibrillation	1121 (33.3)	618 (30.4)	503 (37.7)	<0.001
Cerebral infarction	151 (4.5)	88 (4.3)	63 (4.7)	0.653
Cerebral hemorrhage	47 (1.4)	23 (1.1)	24 (1.8)	0.144
Thrombosis	495 (14.7)	302 (14.9)	193 (14.5)	0.786
Acute respiratory failure	1804 (53.6)	936 (46.1)	868 (65.1)	<0.001
Laboratory examination				
Fibrinogen (mg/dL)	412.6 (230.7)	451.30 (228.60)	353.79 (221.30)	<0.001
WBC, 10^9^/L	14.3 (15.6)	14.11 (16.77)	14.58 (13.75)	0.399
Creatine, mEq/L	3.0 (2.4)	2.74 (2.52)	3.30 (2.19)	<0.001
Hemoglobin, g/dL	10.9 (2.5)	11.06 (2.46)	10.70 (2.48)	<0.001
Albumin, g/dL	2.9 (0.68)	2.97 (0.68)	2.78 (0.71)	<0.001
Platelet, 10^9^/L	195.0 (130.3)	203.21 (131.68)	182.47 (127.19)	<0.001
Potassium, mEq/L	4.4 (1.0)	4.29 (0.98)	4.46 (1.02)	<0.001
Sodium, mEq/L	136.7 (6.7)	136.87 (6.52)	136.43 (7.05)	0.065
Lactate, mmol/L	3.3 (2.8)	2.82 (2.12)	3.98 (3.51)	<0.001
Bilirubin, mg/dL	3.0 (5.9)	2.30 (4.63)	4.06 (7.45)	<0.001
Glucose, mg/dL	150.7 (104.6)	149.60 (109.38)	152.31 (96.79)	0.461
PT, seconds	19.7 (13.5)	18.59 (12.99)	21.57 (14.23)	<0.001
APTT, seconds	37.7 (19.5)	35.52 (16.69)	41.40 (23.03)	<0.001
Score				
SOFA	10.1 (4.8)	8.63 (4.34)	12.29 (4.70)	<0.001
SAPSII	47.7 (16.7)	42.90 (15.05)	54.98 (16.57)	<0.001
Treatment, *n* (%)				
Ventilator	3001 (89.2)	1769 (87.1)	1232 (92.4)	<0.001
RRT	730 (21.7)	415 (20.4)	315 (23.6)	<0.001

**Table 2:** Baseline characteristics of normal fibrinogen and low fibrinogen group before and after matching.

Characteristics	Before matching			After matching		
	Normal fibrinogen (*n* = 1421)	Low fibrinogen (*n* = 374)	*P*	Normal fibrinogen (*n* = 239)	Low fibrinogen (*n* = 239)	*P*
Gender, male, (%)	783 (55.1)	204 (54.5)	0.89	112 (46.9)	127 (53.1)	0.20
Age, mean (SD)	63.62 (16.58)	57.99 (15.13)	<0.001	60.12 (16.13)	60.33 (16.11)	0.89
AKI, *n* (%)	984 (69.2)	311 (83.2)	<0.001	187 (78.2)	192 (80.3)	0.65
COPD, *n* (%)	97 (6.8)	28 (7.5)	0.74	25 (10.5)	20 (8.4)	0.53
CKD, *n* (%)	307 (21.6)	69 (18.4)	0.21	45 (18.8)	48 (20.1)	0.82
Acute respiratory failure, *n* (%)	778 (54.8)	235 (62.8)	0.01	154 (64.4)	145 (60.7)	0.45
Liver cirrhosis, *n* (%)	292 (20.5)	202 (54.0)	<0.001	98 (41.0)	91 (38.1)	0.58
AMI, *n* (%)	88 (6.2)	20 (5.3)	0.63	19 (7.9)	16 (6.7)	0.73
HF, *n* (%)	434 (30.5)	62 (16.6)	<0.001	53 (22.2)	53 (22.2)	1.00
Hypertension, *n* (%)	261 (18.4)	59 (15.8)	0.28	36 (15.1)	37 (15.5)	1.00
Thrombosis, *n* (%)	207 (14.6)	61 (16.3)	0.45	38 (15.9)	48 (20.1)	0.28
AF, *n* (%)	489 (34.4)	80 (21.4)	<0.001	68 (28.5)	65 (27.2)	0.84
Cerebral infarction, n (%)	60 (4.2)	9 (2.4)	0.14	7 (2.9)	7 (2.9)	1.00
Cerebral hemorrhage n (%)	18 (1.3)	4 (1.1)	0.97	3 (1.3)	1 (0.4)	0.62
WBC, mean (SD)	13.83 (11.49)	12.74 (8.85)	0.09	14.63 (13.04)	13.32 (9.58)	0.21
Platelet, mean (SD)	186.34 (123.92)	112.18 (72.78)	<0.001	128.05 (77.83)	131.74 (79.27)	0.61
Creatine, mean (SD)	2.89 (2.34)	3.56 (2.28)	<0.001	3.25 (2.52)	3.40 (2.20)	0.48
Hemoglobin, mean (SD)	10.89 (2.50)	10.49 (2.71)	0.01	10.65 (2.50)	10.73 (2.88)	0.77
Albumin, mean (SD)	2.93 (0.68)	2.74 (0.72)	<0.001	2.78 (0.65)	2.84 (0.74)	0.32
Bilirubin, mean (SD)	3.20 (6.45)	7.80 (9.50)	<0.001	5.74 (9.11)	5.20 (7.50)	0.48
Lactate, mean (SD)	3.40 (2.87)	5.04 (4.26)	<0.001	4.06 (3.37)	4.27 (3.47)	0.49
Potassium, mean (SD)	4.35 (1.01)	4.44 (1.11)	0.14	4.38 (1.08)	4.30 (0.92)	0.39
Sodium, mean (SD)	136.70 (6.64)	134.88 (7.44)	<0.001	135.81 (7.82)	135.59 (7.39)	0.76
PT, mean (SD)	18.91 (11.04)	25.68 (15.35)	<0.001	21.76 (13.62)	21.73 (10.45)	0.98
PTT, mean (SD)	37.65 (19.34)	48.21 (23.79)	<0.001	42.82 (23.96)	42.93 (20.95)	0.96
SOFA score, mean (SD)	10.40 (4.70)	13.90 (4.74)	<0.001	12.44 (4.22)	12.74 (4.73)	0.45
SAPS II score, mean (SD)	48.11 (16.36)	51.73 (17.22)	<0.001	51.13 (16.66)	51.07 (17.78)	0.97
Fibrinogen, mean (SD)	272.95 (72.81)	105.14 (29.54)	<0.001	255.28 (73.59)	106.96 (28.35)	<0.001
Inhospital death, *n* (%)	631 (44.4)	231 (61.8)	<0.001	132 (55.2)	134 (56.1)	0.93

**Table 3:** Baseline characteristics of normal fibrinogen and elevated fibrinogen group before and after matching.

Characteristics	Before matching			After matching		
	Normal fibrinogen (*n* = 1421)	Elevated fibrinogen (*n* = 1570)	*P*	Normal fibrinogen (*n* = 964)	Elevated fibrinogen (*n* = 964)	*P*
Gender, male, (%)	783 (55.1)	893 (56.9)	0.35	532 (55.1)	523 (54.2)	0.72
Age, mean (SD)	63.62 (16.58)	64.30 (16.32)	0.26	64.76 (17.19)	64.57 (16.68)	0.81
AKI, *n* (%)	984 (69.2)	1060 (67.5)	0.33	635 (65.8)	645 (66.8)	0.67
COPD, *n* (%)	97 (6.8)	114 (7.3)	0.70	70 (7.3)	67 (6.9)	0.86
CKD, *n* (%)	307 (21.6)	376 (23.9)	0.14	219 (22.7)	217 (22.5)	0.96
Acute respiratory failure, *n* (%)	778 (54.8)	791 (50.4)	0.019	508 (52.6)	498 (51.6)	0.68
Liver cirrhosis, *n* (%)	292 (20.5)	68 (4.3)	<0.001	51 (5.3)	58 (6.0)	0.55
AMI, *n* (%)	88 (6.2)	89 (5.7)	0.60	62 (6.4)	56 (5.8)	0.64
HF, *n* (%)	434 (30.5)	494 (31.5)	0.61	319 (33.1)	310 (32.1)	0.70
Hypertension, *n* (%)	261 (18.4)	361 (23.0)	0.002	202 (20.9)	199 (20.6)	0.91
Thrombosis, *n* (%)	207 (14.6)	227 (14.5)	0.97	142 (14.7)	150 (15.5)	0.66
AF, *n* (%)	489 (34.4)	552 (35.2)	0.70	341 (35.3)	345 (35.8)	0.89
Cerebral infarction, *n* (%)	60 (4.2)	82 (5.2)	0.23	45 (4.7)	43 (4.5)	0.91
Cerebral hemorrhage, *n* (%)	18 (1.3)	25 (1.6)	0.55	11 (1.1)	13 (1.3)	0.84
WBC, mean (SD)	13.83 (11.49)	15.10 (19.62)	0.033	13.77 (12.26)	13.85 (12.86)	0.89
Platelet, mean (SD)	186.34 (123.92)	222.54 (137.04)	<0.001	200.08 (124.85)	201.86 (127.13)	0.76
Creatine, mean (SD)	2.89 (2.34)	2.88 (2.48)	0.89	2.74 (2.30)	2.72 (2.22)	0.80
Hemoglobin, mean (SD)	10.89 (2.50)	11.05 (2.38)	0.083	11.12 (2.51)	11.01 (2.36)	0.32
Albumin, mean (SD)	2.93 (0.68)	2.91 (0.66)	0.43	2.94 (0.69)	2.95 (0.66)	0.83
Bilirubin, mean (SD)	3.20 (6.45)	1.57 (2.86)	<0.001	1.64 (3.08)	1.78 (3.25)	0.35
Lactate, mean (SD)	3.40 (2.87)	2.75 (2.03)	<0.001	2.95 (2.19)	2.84 (2.07)	0.26
Potassium, mean (SD)	4.35 (1.01)	4.34 (0.96)	0.78	4.32 (0.96)	4.31 (0.94)	0.76
Sodium, mean (SD)	136.70 (6.64)	137.12 (6.58)	0.083	137.25 (6.46)	137.29 (6.49)	0.90
PT, mean (SD)	18.91 (11.04)	18.99 (14.60)	0.88	18.05 (11.04)	18.26 (12.09)	0.69
PTT, mean (SD)	37.65 (19.34)	35.33 (17.59)	0.001	35.85 (17.46)	35.81 (18.62)	0.96
SOFA score, mean (SD)	10.40 (4.70)	8.89 (4.44)	<0.001	9.44 (4.45)	9.26 (4.45)	0.37
SAPSII score, mean (SD)	48.11 (16.36)	46.35 (16.81)	0.004	47.07 (16.15)	46.42 (16.28)	0.38
Fibrinogen, mean (SD)	272.95 (72.81)	612.33 (170.24)	<0.001	283.57 (70.67)	603.98 (166.37)	<0.001
Inhospital death, *n* (%)	631 (44.4)	472 (30.1)	<0.001	387 (40.1)	310 (32.1)	<0.001

**Table 4:** Cox analysis of all included covariates.

	Univariate regression			Multivariate regression		
	HR	95% CI	P	HR	95% CI	P
Age, years						
18-40	—	—	—	—	—	—
40-60	1.73	1.35-2.22	<0.001	1.49	1.15-1.93	0.002
60-80	1.92	1.51-2.44	<0.001	1.98	1.54-2.55	<0.001
>80	2.55	1.97-3.30	<0.001	2.82	2.14-3.72	<0.001
Gender						
Female	—	—	—	—	—	—
Male	0.95	0.85-1.05	0.305	0.90	0.80-1.01	0.064
Comorbidities (patients without comorbidities as baseline)						
AMI	1.48	1.21-1.81	<0.001	1.17	0.95-1.43	0.15
Heart failure	1.14	1.01-1.27	0.03	0.97	0.86-1.11	0.69
Liver cirrhosis	1.70	1.50-1.94	<0.001	1.11	0.94-1.31	0.21
Hypertension	1.00	0.87-1.14	0.95	1.08	0.93-1.25	0.32
COPD	1.31	1.08-1.59	0.006	1.16	0.95-1.42	0.14
CKD	1.17	1.03-1.32	0.016	0.89	0.77-1.02	0.097
Atrial fibrillation	1.24	1.11-1.38	<0.001	1.01	0.90-1.15	0.85
Cerebral infarction	0.97	0.75-1.25	0.82	0.98	0.76-1.28	0.89
Cerebral hemorrhage	1.36	0.94-2.03	0.14	1.83	1.21-2.78	0.005
Thrombosis	0.91	0.78-1.06	0.24	0.90	0.77-1.05	0.17
Acute respiratory failure	1.80	1.61-2.01	<0.001	1.53	1.35-1.72	<0.001
AKI	1.98	1.73-2.26	<0.001	1.19	1.00-1.41	0.047
Laboratory examination (patients within normal range as reference)						
Fibrinogen						
<150 mg/dL	1.66	1.42-1.93	<0.001	1.22	1.03-1.44	0.022
>400 mg/dL	0.60	0.53-0.68	<0.001	0.66	0.59-0.75	<0.001
WBC, 10^9^/L						
<4	1.12	0.92-1.35	0.27	1.24	1.02-1.51	0.034
>11	1.09	0.97-1.22	0.17	1.06	0.94-1.20	0.31
Creatine, mEq/L	1.06	1.04-1.08	<0.001	1.50	1.20-1.88	<0.001
Hemoglobin, g/dL	1.29	1.15-1.46	<0.001	1.26	1.11-1.44	<0.001
Albumin, g/dL	0.72	0.66-0.77	<0.001	0.84	0.61-1.16	0.29
Platelet, 10^9^/L						
<150	1.31	1.17-1.46	<0.001	0.71	0.37-1.38	0.31
>400	0.65	0.65-1.05	0.12	1.10	0.96-1.27	0.18
Potassium, mEq/L						
<3.3	0.94	0.80-1.10	0.41	0.94	0.80-1.11	0.49
>5.1	1.33	1.16-1.51	<0.001	1.11	0.97-1.28	0.12
Sodium, mEq/L						
<133	1.21	1.08-1.35	0.001	1.15	1.03-1.30	0.016
>145	1.48	1.17-1.88	0.001	1.32	1.04-1.70	0.026
Lactate, mmol/L	1.13	1.11-1.16	<0.001	1.33	1.18-1.50	<0.001
Bilirubin, mg/dL	1.03	1.03-1.04	<0.001	1.09	0.96-1.25	0.19
Glucose, mg/dL						
<70	1.81	1.43-2.30	<0.001	1.42	1.12-1.80	0.004
>105	0.96	0.85-1.10	0.54	0.99	0.87-1.13	0.87
PT, seconds						
<10.4	0.65	0.33-1.26	0.20	0.71	0.37-1.38	0.31
>13.4	1.45	1.28-1.65	<0.001	1.10	0.96-1.27	0.18
APTT, seconds						
<22	1.34	0.91-1.96	0.14	1.30	0.88-1.92	0.19
>35	1.74	1.57-1.95	<0.001	1.35	1.20-1.52	<0.001
Treatment						
Ventilator	1.61	1.31-1.97	<0.001	1.10	0.89-1.36	0.40
RRT	1.80	1.60-2.02	<0.001	1.43	1.26-1.63	<0.001

**Table 5:** Aggregation of Cox regression model.

Model	Low level (<150 mg/dL)		High level (>400 mg/dL)	
	HR (95% CI)	*P* value	HR (95% CI)	*P* value
Unadjusted	1.66 (1.42, 1.93)	<0.001	0.60 (0.53, 0.68)	<0.001
Model 1	1.79 (1.54, 2.09)	<0.001	0.59 (0.53, 0.67)	<0.001
Model 2	1.50 (1.28, 1.76	<0.001	0.64 (0.56, 0.72)	<0.001
Model 3	1.22 (1.03, 1.44)	0.022	0.66 (0.59, 0.75)	<0.001

The reference group was the normal fibrinogen group (150-400 mg/dL). Model 1 adjusted for age and gender. Model 2 adjusted for model 1 covariates+AKI, AMI, HF, liver cirrhosis, hypertension, COPD, CKD, AF, cerebral infarction, cerebral hemorrhage, thrombosis, and acute respiratory failure. Model 3 adjusts for model 2+WBC, Scr, hemoglobin, albumin, platelet, blood potassium, sodium, lactate, total bilirubin, glucose, prothrombin time (PT), and partial thromboplastin time (PTT), use of ventilation and RRT.

**Table 6:** Subgroup analysis of the association between fibrinogen and inhospital mortality based on model 3.

Characteristics	N	Low fibrinogen HR (95% CI)	*P*	Elevated fibrinogen HR (95% CI)	*P*	*P* for interaction
Age						0.43
<40	307	1.20 (0.61, 2.37)	0.60	0.71 (0.39, 1.32)	0.29	
40-59	965	1.47 (1.12, 1.93)	0.006	0.68 (0.51, 0.90)	0.007	
60-79	1543	1.05 (0.81, 1.38)	0.70	0.60 (0.50, 0.72)	<0.001	
≥80	547	1.49 (0.91, 2.43)	0.11	0.85 (0.65, 1.11)	0.22	
Gender						0.33
Female	1484	1.19 (0.93, 1.52)	0.17	0.61 (0.51, 0.74)	<0.001	
Male	1878	1.25 (0.99, 1.58)	0.056	0.70 (0.59, 0.83)	<0.001	
AMI						0.068
No	3165	1.23 (1.04, 1.46)	0.018	0.64 (0.56, 0.73)	<0.001	
Yes	197	1.48 (0.71, 3.09)	0.29	1.01 (0.61, 1.67)	0.98	
Heart failure						0.58
No	2372	1.18 (0.98, 1.43)	0.08	0.66 (0.56, 0.77)	<0.001	
Yes	990	1.26 (0.86, 1.83)	0.23	0.64 (0.52, 0.80)	<0.001	
Liver cirrhosis						0.88
No	2800	1.18 (0.94, 1.48)	0.16	0.66 (0.58, 0.75)	<0.001	
Yes	562	1.31 (1.00, 1.71)	0.048	0.76 (0.48, 1.21)	0.25	
Hypertension						0.88
No	2683	1.22 (1.02, 1.47)	0.031	0.65 (0.56, 0.74)	<0.001	
Yes	679	1.26 (0.82, 1.94)	0.29	0.70 (0.53, 0.93)	0.013	
COPD						0.63
No	3123	1.23 (1.03, 1.46)	0.019	0.67 (0.59, 0.77)	<0.001	
Yes	239	1.11 (0.56, 2.22)	0.77	0.60 (0.373, 0.95)	0.029	
CKD						0.92
No	2610	1.21 (1.00, 1.45)	0.049	0.67 (0.57, 0.77)	<0.001	
Yes	752	1.33 (0.90, 1.98)	0.16	0.66 (0.52, 0.84)	0.001	
Atrial fibrillation						0.004
No	2241	1.22 (1.01, 1.49)	0.044	0.58 (0.49, 0.69)	<0.001	
Yes	1121	1.07 (0.76, 1.51)	0.69	0.81 (0.67, 0.98)	0.028	
Cerebral infarction						0.001
No	3211	1.18 (1.00, 1.40)	0.053	0.63 (0.55, 0.72)	<0.001	
Yes	151	6.90 (1.94, 24.51)	0.003	1.73 (0.87, 3.47)	0.12	
Cerebral hemorrhage						0.59
No	3315	1.21 (1.03, 1.44)	0.024	0.67 (0.59, 0.76)	<0.001	
Yes	47	5.25 (0.03.987.47)	0.54	0.21 (0.001, 55.66)	0.58	
Thrombosis						0.13
No	2867	1.27 (1.06, 1.53)	0.009	0.68 (0.60, 0.78)	<0.001	
Yes	495	0.90 (0.56, 1.45)	0.66	0.60 (0.43, 0.84)	0.003	
Acute respiratory failure						0.004
No	1561	1.39 (1.04, 1.87)	0.029	0.52 (0.42, 0.64)	<0.001	
Yes	1801	1.15 (0.94, 1.41)	0.19	0.76 (0.65, 0.88)	<0.001	
Ventilator						0.103
No	364	2.43 (1.18, 5.02)	0.016	0.45 (0.28, 0.73)	0.001	
Yes	2998	1.17 (0.99, 1.40)	0.073	0.68 (0.60, 0.78)	<0.001	
RRT						0.33
No	2633	1.30 (1.05, 1.61)	0.016	0.68 (0.59, 0.79)	<0.001	
Yes	729	1.02 (0.78, 1.34)	0.89	0.59 (0.46, 0.75)	<0.001	
AKI						0.18
No	1009	1.80 (1.15, 2.81)	0.01	0.63 (0.48, 0.84)	0.001	
Yes	2353	1.18 (0.98, 1.41)	0.078	0.94 (0.65, 1.35)	<0.001	
WBC, 10^9^/L						<0.001
4-11	1236	1.11 (0.84, 1.49)	0.47	0.53 (0.42, 0.66)	<0.001	
< 4	323	1.11 (0.57, 2.15)	0.75	1.52 (0.98, 2.36)	0.061	
> 11	1803	1.32 (1.05, 1.67)	0.019	0.65 (0.50, 1.08)	<0.001	
Creatine, mEq/L						0.94
< 1.2	676	2.09 (1.04, 4.21)	0.039	0.62 (0.43, 0.90)	0.011	
≥ 1.2	2686	1.20 (1.01, 1.43)	0.037	0.67 (0.59, 0.77)	<0.001	
Hemoglobin, g/dL						0.29
< 12	1122	1.34 (0.97, 1.85)	0.072	0.66 (0.53, 0.83)	<0.001	
≥ 12	2240	1.17 (0.96, 1.43)	0.11	0.66 (0.57, 0.77)	<0.001	
Albumin, g/dL						0.50
< 3.5	114	0.81 (0.21, 3.16)	0.76	0.41 (0.16, 1.06)	0.067	
≥ 3.5	3248	1.20 (1.02, 1.43)	0.033	0.67 (0.59, 0.76)	<0.001	
Platelet, 10^9^/L						0.07
150-400	1724	1.21 (0.88, 1.67)	0.24	0.73 (0.62, 0.87)	<0.001	
< 150	1399	1.15 (0.94, 1.42)	0.18	0.53 (0.43, 0.66)	<0.001	
> 400	239	24.88 (1.94, 319.14)	0.014	1.13 (0.60, 2.11)	0.707	
Potassium, mEq/L						0.81
3.3-5.1	2198	1.13 (0.91, 1.40)	0.26	0.66 (0.57, 0.78)	<0.001	
< 3.3	500	1.32 (0.86, 2.03)	0.21	0.71 (0.48, 1.04)	0.077	
> 5.1	664	1.37 (0.95, 1.99)	0.094	0.64 (0.48, 0.84)	0.001	
Sodium, mEq/L						0.45
133-145	1906	1.13 (0.88, 1.45)	0.35	0.68 (0.57, 0.80)	<0.001	
< 133	1305	1.21 (0.96, 1.54)	0.11	0.60 (0.49, 0.74)	<0.001	
> 145	151	2.35 (0.81, 6.84)	0.12	0.35 (0.16, 0.76)	0.007	
Lactate, mmol/L						0.71
< 2	1292	1.31 (0.91, 1.89)	0.15	0.65 (0.52, 0.80)	<0.001	
> 2	2070	1.15 (0.95, 1.40)	0.15	0.67 (0.58, 0.79)	<0.001	
Bilirubin, mg/dL						0.002
< 1.5	2124	1.01 (0.73, 1.40)	0.95	0.74 (0.64, 0.86)	<0.001	
≥ 1.5	1238	1.21 (0.99, 1.49)	0.068	0.52 (0.41, 0.66)	<0.001	
Glucose, mg/dL						0.72
70-105	843	1.09 (0.78, 1.52)	0.62	0.67 (0.52, 0.88)	0.003	
< 70	150	1.44 (0.78, 2.65)	0.25	0.85 (0.40, 1.81)	0.68	
> 105	2369	1.29 (1.05, 1.60)	0.018	0.8 (0.58, 0.78)	<0.001	
PT, seconds						0.38
10.4-13.4	963	1.38 (0.85, 2.23)	0.19	0.81 (0.64, 1.02)	0.078	
< 10.4	41					
> 13.4	2358	1.19 (0.99, 1.42)	0.063	0.61 (0.53, 0.71)	<0.001	
APTT, seconds						0.44
22-35	2049	1.42 (1.07, 1.88)	0.016	0.69 (0.59, 0.81)	<0.001	
< 22	63	—	—	—	—	
> 35	1250	1.12 (0.91, 1.39)	0.28	0.61 (0.49, 0.74)	<0.001	

## Data Availability

Publicly available datasets were used in this study. These can be found in MIMIC-IV at doi:10.13026/s6n6-xd98.

## Supplementary Materials

Supplementary Materials A summary comparing Cox regressions before and after PSM can be seen in Supplementary Table 1.
