# Supplementary material for: Fibrinogen Is Associated with Prognosis of Critically Ill Patients with Sepsis: A Study Based on Cox Regression and Propensity Score Matching
Source: Mediators Inflamm. 2023 Mar 20;2023:7312822. doi: 10.1155/2023/7312822 (PMC10042635; doi:10.1155/2023/7312822)
Supplement: Supplementary Materials — A summary comparing Cox regressions before and after PSM can be seen in Supplementary Table 1. [file 7312822.f1.docx]

Table A.1 Cox regression of decreased level of fibrinogen

|  |  | univariate Cox | multivariate Cox | univariate logistic | multivariate logistic |
| --- | --- | --- | --- | --- | --- |
| Before PSM (N=3365) | fibrinogen level (categorical data) | 1.66 (1.42, 1.93), P<0.001 | 1.22 (1.03, 1.44), P=0.022 | 2.02 (1.60, 2.55), P<0.001 | 1.32 (1.00, 1.75), P=0.051 |
|  | fibrinogen  (continuous data) | 1.00 (1.00, 1.00), P<0.001 | 1.00 (1.00, 1.00), P<0.001 | 1.00 (1.00, 1.00), P<0.001 | 1.00 (1.00, 1.00)  ,P<0.001 |
| After PSM (N=478) | fibrinogen level (categorical data) | 1.00 [0.79, 1.28], P=0.98 | - | 1.03 [0.72, 1.48], P=0.85 | - |
|  | fibrinogen  (continuous data) | 1.00 [1.00, 1.00], P=0.22 | - | 1.00 [1.00, 1.00], P=0.19 | - |

Tip: We have had a concise description of the supplementary files inside the manuscript file. Please refer to line 202-203 in discussion section.
